# Management of epidermal cysts arising from scar tissues: A retrospective clinical study

**DOI:** 10.1097/MD.0000000000012188

**Published:** 2018-08-21

**Authors:** Hae Woong Lee, Chang Gyun Kim, Ji Sun Song, In Chang Koh, Hoon Kim, Kyu Nam Kim

**Affiliations:** a Department of Dermatology, Louis Dermatologic Clinic, Guri; b Department of Plastic and Reconstructive Surgery, Konyang University Hospital, University of Konyang College of Medicine, Myunggok Medical Research Center, Daejeon; c Department of Plastic and Reconstructive Surgery, Catholic Kwandong University, International St. Mary's Hospital, Incheon, Korea.

**Keywords:** dermatology, epidermal cyst, keloid, lasers, scars

## Abstract

Few reports have described epidermal cysts (ECs) arising from scar tissues, and the standard course of treatment has not been established. We aimed to report the findings of a Korean patient series with ECs arising from scar tissues, to describe patient management in the context of previous publications, and to present a simple algorithm for managing ECs arising from scar tissues.

We managed 6 patients with ECs arising from scar tissues, and retrospectively reviewed their demographic and clinical data.

The scars were located on the anterior chest wall (n = 3), shoulder (n = 1), forehead (n = 1), and ear lobule (n = 1). Two patients with anterior chest wall scars, 1 with a shoulder scar, and 1 with an ear lobule scar had keloid scars, whereas the other patients had hypertrophic scars. The scar sizes ranged from 2 × 1 cm to 9 × 7 cm. The EC sizes ranged from 0.2 × 0.2 cm to 2 × 1.5 cm. Three patients underwent total scar revisions with complete EC excisions, 2 underwent partial scar tissue excisions with complete EC excisions, and 1 had laser therapy for the scar and EC. No complications occurred, and all patients’ final outcomes were satisfactory during the mean follow-up period of 14.8 months.

We successfully managed the patients with ECs arising from scar tissues. We recommend that surgeons and patients first decide whether the ECs and scar tissue should be completely removed. Moreover, consideration should be given to the options chosen for the management of ECs. Finally, postoperative scar care is necessary to prevent hypertrophic and keloid scar recurrences.

## 1 Introduction

Epidermal cysts (ECs) are slow growing, elevated, round, firm, and intradermal or subcutaneous lesions that occur most commonly on the face, scalp, neck, and trunk.^[1]^ Historically, ECs have been called follicular infundibular cysts, epidermal inclusion cysts, and epidermoid cysts.^[2]^ The treatment of ECs varies depending on the size, location, and whether an infection is present; treatment includes the puncture and aspiration method, intralesional triamcinolone injections for infection control, and surgical excision.^[3–6]^ The management of ECs that occur in normal skin tissue is fairly straightforward. However, the management of ECs arising from scar tissues, such as hypertrophic scars and keloids, should be approached differently, because scar tissues are vulnerable to wound-healing processes and scars are likely to recur recurrence. Moreover, we consider that the occurrence of ECs arising from scar tissues is theoretically unusual, because scar tissues do not contain epidermal appendages.

Only a few reports have described ECs arising from scar tissues, and the management of this condition has not been established yet. The aim of this study was to describe the ECs arising from scar tissues in 6 Korean patients and to present an appropriate management algorithm developed in the context of previous publications.

## 2 Methods

All procedures and assessments described in this report were approved by the institutional ethics review board of Konyang University Hospital (Approval number: KUH 2017-01-004), and were conducted in accordance with the 1964 Helsinki Declaration and its later amendments or comparable ethical standards. All patients provided written informed consent.

Between April 2014 and April 2017, we managed 6 patients, comprising 4 men and 2 women with a mean age of 37 years (range 22–55 years), who presented with ECs arising from scar tissues. The ECs had developed after scar tissue had formed in the patients. None of the patients had a history of trauma within the area of the scar tissue. We retrospectively reviewed each patient's medical charts, and recorded the location, cause, size, and type of the scar, the size of the EC, and the patient's management, complications, and follow-up duration.

## 3 Results

Table 1 presents the patients’ characteristics and clinical data. The scars were formed due to trauma in 3 patients, a surgical incision in 1 patient, piercing in one patient, and post-Bacillus Calmette–Guérin vaccination in 1 patient. The scars were located on the anterior chest wall *(*n = 3), shoulder (n = 1), forehead (n = 1), and ear lobule (n = 1). Keloid scars were found in 2 patients with anterior chest wall scars, 1 with an ear lobule scar, and 1 with a shoulder scar had keloid formation, whereas hypertrophic scars were observed in 1 patient with an anterior chest wall scar and 1 with a forehead scar. The sizes of the scars ranged from 2 × 1 cm to 9 × 7 cm, and those of the ECs ranged from 0.2 × 0.2 cm to 2 × 1.5 cm. ECs of 5 patients did not rupture, whereas the EC of 1 patient did.

**Table 1:**
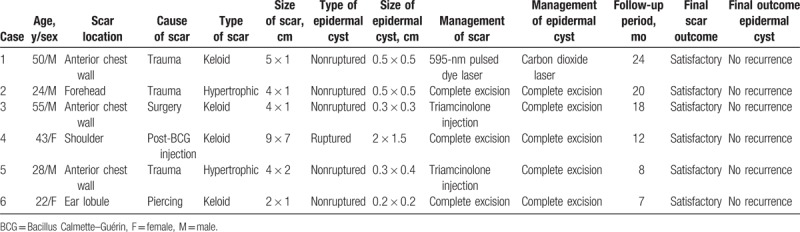
Summary of the patients’ characteristics.

Regarding the management of the scars, 3 patients underwent complete excisions (total scar revision), 1 had laser therapy, and 2 were administered triamcinolone injections. All the patients underwent subsequent scar care that included application of taping fixation and silicone gel sheets, and use of compression garments. In relation to the management of the ECs, 5 patients underwent complete excisions and 1 had laser therapy. The patients had no complications, such as delayed wound healing, EC recurrence, and scar aggravation. The follow-up periods ranged from 7 to 24 months (mean 14.8 months), and all patients were satisfied with the final outcomes.

## 4 Case reports

### 4.1 Case 1

**Figure 1:**
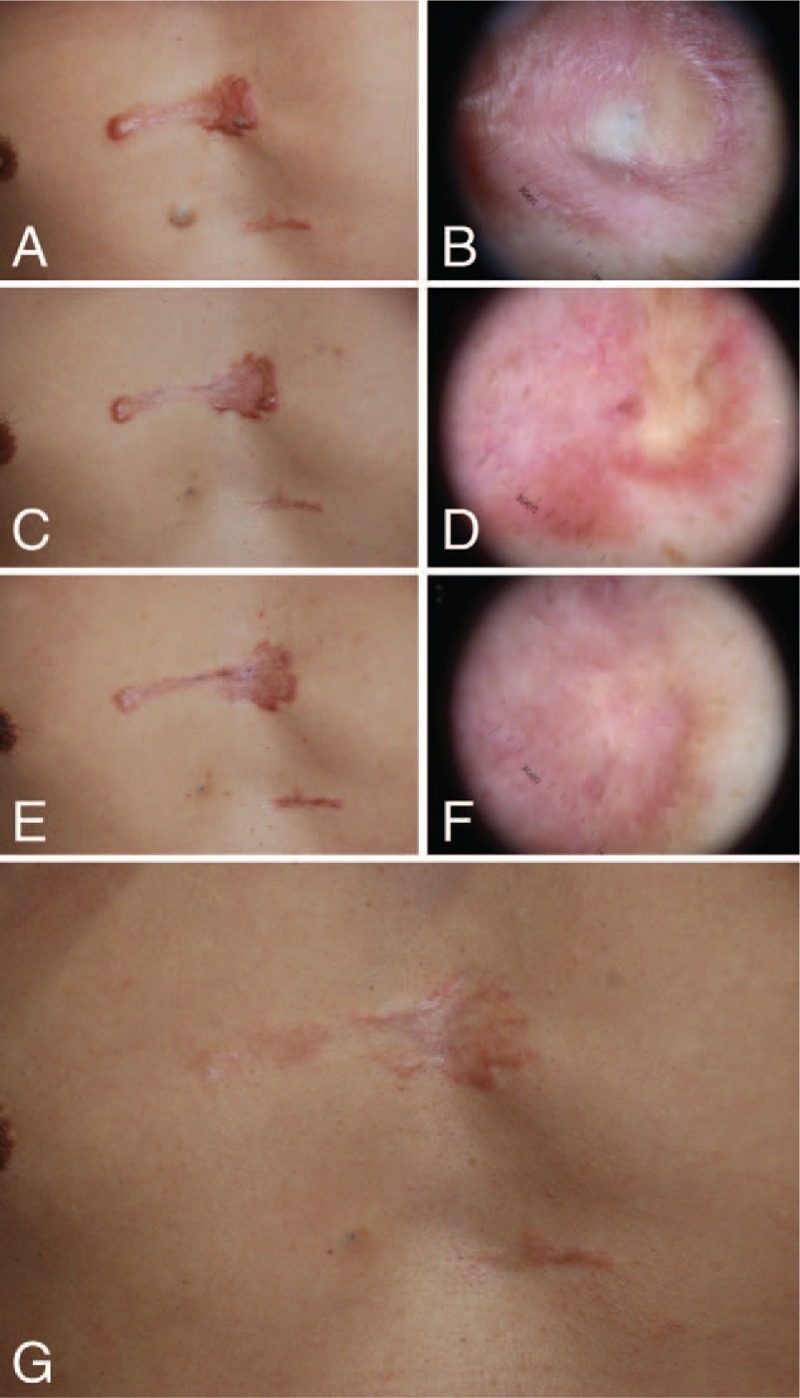
A 50-year-old man presented with an epidermal cyst arising from a keloid scar and underwent laser therapy (Case 1). (A) The keloid scar with a left-sided cystic lesion on the anterior chest wall. (B) The dermoscopic examination reveals an epidermal cyst with a central skin opening. (C) Image obtained at one month postoperatively. (D) The dermoscopic findings at 1 month postoperatively. (E) Image obtained at 9 months postoperatively. (F) The dermoscopic findings at 9 months postoperatively. (G) Image obtained at 2 years postoperatively.

A 50-year-old Korean man presented with a keloid scar on his anterior chest wall, which had developed 1 year prior following trauma and had gradually enlarged beyond the original wound's boundaries. He complained about pain and itching sensations in the keloid scar. The keloid scar measured about 5 cm and was located on the sternal area; a cystic lesion with a pin-point-sized skin opening was present at the center of the keloid scar (Fig. 1A). The cystic lesion was absent before the traumatic event, and dermoscopic examination revealed an EC with a central skin opening (Fig. 1B). The patient was asked to choose which among the 2 treatment options to undergo: total excision of the scar tissue including the cyst or laser therapy for the keloid and cystic lesion; the patient chose the latter method. First, laser therapy using a 595-nm pulsed dye laser (Vbeam; Candela Corporation, Wayland, MA) with a 7-mm spot size at a fluency of 12 J/cm^2^ and a 20-ms pulse width was performed to manage the keloid scar. Adjacent, nonoverlapping laser pulses were applied to the entire surface of the keloid scar. Epidermal cooling was achieved using a cryogen spray cooling device, which had a spurt duration of 30 ms with a delay of 30 ms. These procedures were performed 3 times with 3-week intervals. Second, laser therapy using a carbon dioxide laser (Spectra SP carbon dioxide laser, 20 Hz, 250 μs pulse width, 17 mJ; Lutronic, Korea) to create multiple punctures, resulting in a 2 to 4 mm hole, was performed to manage the EC. Gentle pressure and squeezing led to the exudation of the cyst's internal contents through the hole, and the remaining visible cystic walls were cauterized using the carbon dioxide laser. We applied Steri-Strip skin closures (3 M, Maplewood, MN) to the wound without surgical sutures. There were no postoperative complications, such as infection or wound dehiscence. The EC healed completely following the carbon dioxide laser therapy. We performed 5 further pulsed dye laser cycles on the whole keloid scar (Fig. 1C and D). After completing the laser therapy, Mepiform dressing (Mölnlycke Health Care, Oakville, Ontario, Canada), which is a self-adherent soft silicone dressing designed for scar management, was used by the patient for 5 months (Fig. 1E and F). There were no EC recurrences or keloid overgrowth during the 2-year follow-up period, and the patient was satisfied with the final outcome (Fig. 1G).

### 4.2 Case 2

**Figure 2:**
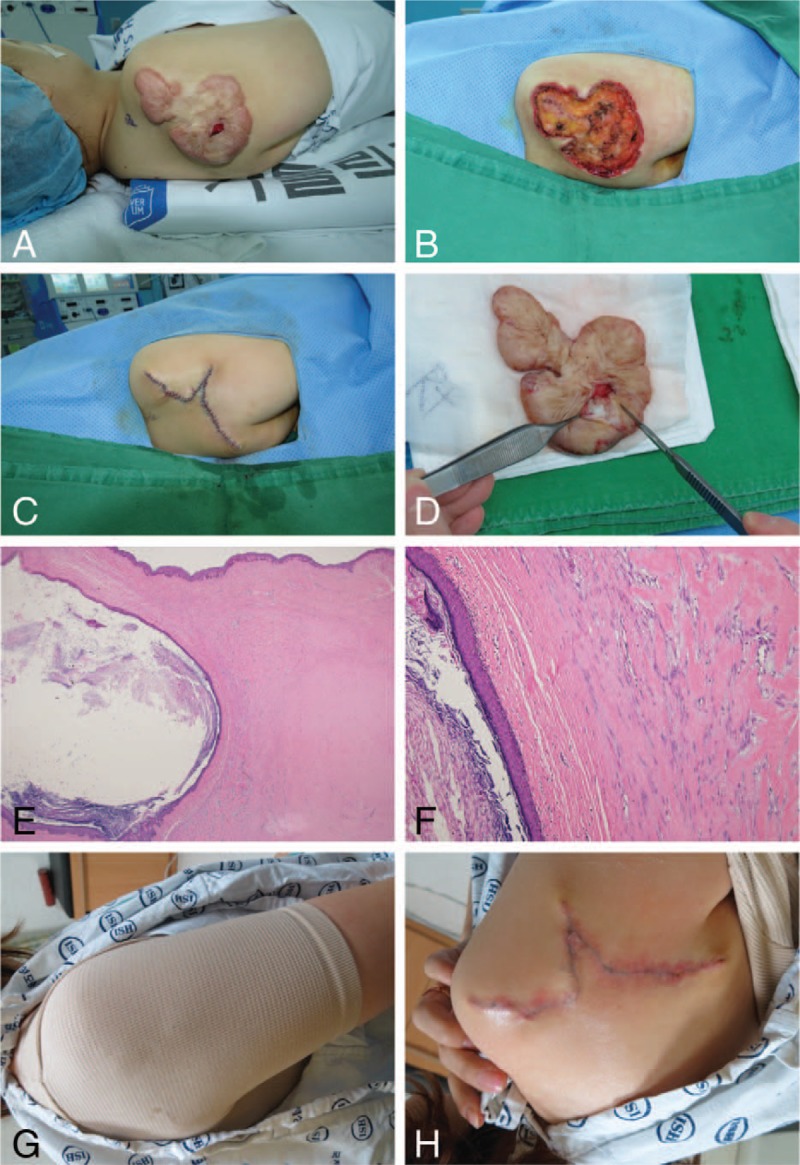
A 43-year-old woman presented with an epidermal cyst arising from a keloid scar and underwent surgical excision (Case 4). (A) The keloid with a centrally located ruptured cystic lesion on the right shoulder. (B) Total excision of the keloid tissue, including the cystic lesion. (C) Immediate postoperative image. (D) Keratinaceous material within the cyst. (E) Histopathologically, a multilayered keratin-filled cyst (left-side) and a dense collagenous keloid (right-side) are present in the dermis in a low-power view. The cyst wall comprised a stratified squamous epithelium with a granular layer (hematoxylin and eosin ×12.5). (F) A high-powered histopathologic view shows that the adjacent dermis contained characteristic broad, eosinophilic, and homogeneous keloidal collagen bundles (hematoxylin and eosin ×100). (G) The patient applies a personalized compression garment for 5 months. (H) Image obtained at 3 months postoperatively.

A 43-year-old woman presented with post-Bacillus Calmette–Guérin vaccination keloid scars on both shoulders. A protruding lesion had developed at the center of the keloid scar on her right shoulder 3 months prior. The lesion had increased in size, and she had squeezed out the lesion's contents several times. However, the lesion had become swollen again and was painful. When she visited our clinic, the lesion had already ruptured, and inflammation had spread to the surrounding keloid scar tissue. The keloid scar on the patient's right shoulder measured about 9 × 7 cm, and the ruptured lesion measured 2 × 1.5 cm (Fig. 2A). We excised all the keloid tissue including the ruptured lesion, and repaired the wound using subdermal 3-0 PDS (Ethicon, Inc., Somerville, NJ) and interrupted 5-0 Ethilone (Ethicon, Inc.) sutures, primarily (Fig. 2B–D). Histopathologically, a large laminated keratin-filled cyst was present in the dermis surrounded with a dense collagenous keloid scar (Fig. 2E). The cyst wall consisted of stratified squamous epithelium with a granular layer, which was consistent with an EC. The adjacent dermis contained characteristic broad, eosinophilic, and homogeneous keloidal collagen bundles (Fig. 2F). There were no postoperative complications, such as an infection or wound dehiscence. The stitches were removed 14 days postoperatively, and Steri-Strip skin closures were applied for 1 month to prevent wound dehiscence and scar widening. Subsequently, the patient used Mepiform and applied a personalized compression garment for 5 months (Fig. 2G and H). There were no EC recurrences or keloid overgrowth during the 1-year follow-up period, and the patient was satisfied with the final outcome.

## 5 Discussion

ECs are unilocular cysts without septation that are encapsulated by fibrous tissues and lined by the true epidermis, which comprises stratified and keratinized squamous epithelium with a granular layer, similar to that found on the skin's surface and in the infundibula of hair follicles.^[7]^ The cysts contain central, eosinophilic, keratinaceous material that comprises accumulated cutaneous products, keratin debris, proteins, cholesterol, and cell membrane lipids.^[1,2,7]^ The contents of ECs usually have a cheesy consistency and are foul smelling.^[7]^ Cysts can be yellow, white, or may have a similar color to the surrounding skin, usually increase in size gradually, and are asymptomatic.^[7]^ However, polymicrobial infections of cysts by aerobic or anaerobic organisms may occur. Furthermore, malignant transformation, despite very rarely, has been reported. In particular, a recent review of the literature has found 41 well-documented cases of squamous cell carcinoma (SCC) arising from cutaneous ECs.^[8]^ The development of SCC is likely to be higher in the case of ECs arising from scar tissues due to a local immune destabilization.^[9,10]^

The term “epidermal inclusion cyst” refers specifically to an EC that is a consequence of the implantation of epidermal elements into the dermis.^[1,2]^ Therefore, epidermal inclusion cysts are ECs of traumatic origins that are more common in nonfollicular areas of the skin, such as the palms, soles, or buttocks.^[7,11]^ The mechanisms that may underlie the development of ECs include the incomplete cleavage of the cutaneous ectoderm at the embryonic stage, squamous metaplasia of the columnar epithelial cells within dilated ducts, the downward growth of the epidermal cells accompanied by inflammation following the obstruction of a hair follicle, human papilloma virus infection,^[12,13]^ or the growth of implanted epidermal fragments within the deep tissue following trauma or surgical procedures.^[1,2,7,8]^ ECs of nontraumatic origins are commonly located in hair-bearing areas of the upper chest, upper back, neck, or head, because most of these lesions originate from the follicular infundibulum.^[7]^

**Figure 3:**
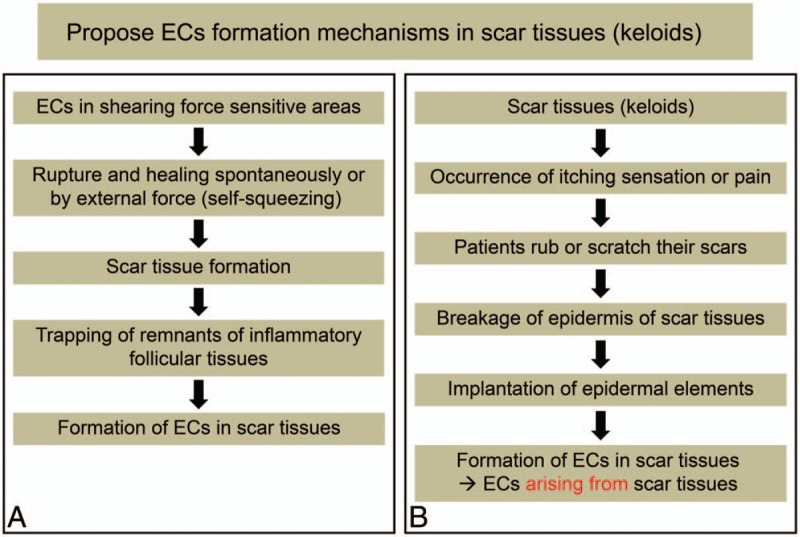
Proposed epidermal cyst (EC) formation mechanisms in scar tissues (keloid) (A) ECs in shearing force sensitive areas can result in scar tissues (keloids). Remnants of inflammatory follicular tissues can be trapped in these scar tissues, which results in the formation of ECs in scar tissues (keloid). (B) Scar tissues (keloid) can cause an itching sensation or pain. Therefore, patients rub or scratch their scars, which breaks the epidermis of the scar tissues and epidermal elements become implanted in the dermis, leading to the development of ECs within the scar tissues.

A mature scar, which is the final product of normal wound healing, is characterized by its disorganized array of collagen and the loss of dermal appendages.^[14]^ Given that there are no epidermal appendages, such as hair follicles and sebaceous glands, in scar tissues, ECs arising from scar tissues may be of traumatic origins rather than nontraumatic origins. ECs are entities that can result in keloid scar formation in shearing force sensitive areas of the body, such as the chest, jaw lines, shoulders, arms, thighs, and knees. Remnants of inflammatory follicular tissues, traumatic or nontraumatic, can be trapped in these scar tissues, providing a source for the inflammation resulting in the formation of ECs in scar tissue (keloid) (Fig. 3A). This condition may be more frequent among patients with Asian ethnicity, because they are more prone to keloid scar formation. However, none of the patients in our study had a history of ECs prior to the scar formation and had no history of trauma associated with their scars. The patients only complained of itching sensations in the regions where the scars were located, and rubbing or scratching their scars to relieve the itching resulted in breakage of the epidermis; hence, epidermal elements were implanted in the dermis, leading to the formation of ECs within the scar tissues (Fig. 3B).

The surgical treatment options for ECs generally include puncture and aspiration, minimal excision surgery, and total excision surgery. Puncture and aspiration and minimal excision surgery are suitable for small cysts and cause minimal scarring, but they carry a risk of recurrence.^[15–17]^ Total excision surgery can remove the cyst completely without the risk of recurrence, but a large scar may form.^[17]^ In relation to the infected ECs, infection control should be considered a priority, which includes administration of oral antibiotics and performing an incision and drainage, if needed. However, regarding ECs arising from scar tissues, it should be considered that scar tissues are vulnerable to wound-healing processes; hence, scars are likely to recur. Total scar revision, involving the complete excision of both the scar tissue and EC, is the primary solution for the removal of both ECs and scars. Three of the patients described in this report wanted both the scars and the ECs removed; thus, we performed a total scar revision surgery. Sometimes, patients may only prefer the EC removal or they may request for EC and scar removal without undergoing a surgery. In both of these situations, the complete excision of the EC may be difficult because of hindrances relating to the wound-healing process and scar tissue growth, particularly in relation to keloid scars. Laser therapy was applied to both the keloid and the cystic lesion of one of the cases described in this report, which involved using a 595-nm pulsed dye laser to remove the keloid, followed by the application of a carbon dioxide laser to remove the cyst's wall. Laser therapy enables the straightforward manipulation and removal of the cyst wall, provides a clear view, and minimizes the scar size associated with the cystic lesion.^[17]^ Previous studies have described the efficacy of laser therapy, including carbon dioxide laser and erbium:yttrium aluminum garnet laser, in epithelial cysts,^[17–19]^ which can be a good alternative for the eradication of uninfected cysts, especially large cysts or cysts located in areas with thicker skin, or for those patients concerned with the cosmetic outcome.^[17]^ In general, the procedures of laser therapy for cystic lesion are as follows. First, multiple fenestrations, creating a hole of about 2 to 4 mm in diameter, are made by a laser device.^[17–19]^ Second, the cyst contents are extracted manually through the hole by gentle digital compression.^[17–19]^ Third, the remaining cystic wall is either removed by careful curettage or ablated by laser.^[17–19]^ Moreover, pulsed dye lasers are effective at improving the texture of hypertrophic and keloid scars, as well as their redness, size, and pliability.^[20,21]^ Long-pulsed dye laser therapy and intense pulsed light are also effective at improving the appearance of hypertrophic and keloids scars.^[21]^ Two patients described in this report only wanted the EC removed without total scar revision surgery, because of the financial burden associated with the procedures. Korea's national health insurance system can cover EC treatment, but it does not cover the scar treatment. Thus, some patients who cannot afford the treatment often only undergo procedures that are covered by the national health insurance system. Consequently, we only performed partial excisions of the scar tissue, which included the ECs, on these 2 patients, and administered intralesional triamcinolone injections to the remaining scarred areas.

Postoperative scar management is important to prevent the recurrence of hypertrophic and keloid scars. Our scar management regimen included the use of taping fixation with nonstretch microporous tape for 1 month postoperatively and silicone-based therapy, which comprised application of silicone gel sheets and wearing of compression garments for 5 months postoperatively. Nonstretch microporous tape is inflexible and provides good scar support,^[21]^ and it may reduce hypertrophic scarring by mimicking the corneum and accelerating healing.^[21,22]^ The occlusion that is achieved using silicone gel sheets reduces scar hypertrophy and the tension in and stability of scars.^[21,23]^ Previous studies’ findings have shown that silicone gel sheets are also effective at reducing the thickening, pain, itching, and pliability associated with severe hypertrophic scars.^[24]^ The findings from most of the clinical trials conducted using silicone gel sheets have confirmed the efficacy and safety of this treatment modality in scar management.^[21,24–27]^ None of our patients had scar recurrences during follow-up.

Our study has some limitations. First, we included a very small number of heterogenous cases and used a nonrandomized, retrospective design with no comparison group. Thus, further prospective large-scale studies are required to overcome selection biases and confounding factors, and to confirm the consistently favorable results. Second, not all cyst cases were pathologically confirmed. We could not confirm the EC histopathologically in Case 1, because the patient did not want further diagnostic evaluation; thus, we performed laser ablation of the cyst immediately. However, the EC can generally be diagnosed based on clinical and physical findings.^[28]^ Moreover, recent studies concluded that the identification of the punctum by dermoscopy, a pore corresponding to the follicle from which the cyst is derived, gives a clue to the diagnosis.^[29,30]^ The presence of a pore in a subcutaneous nodule allows the diagnosis of EC to be made.^[29]^ In Case 1 of this study, our dermatologist (Hae Woong Lee, MD) found the “pore” sign using dermoscopic examination and diagnosed it clinically as the EC.

**Figure 4:**
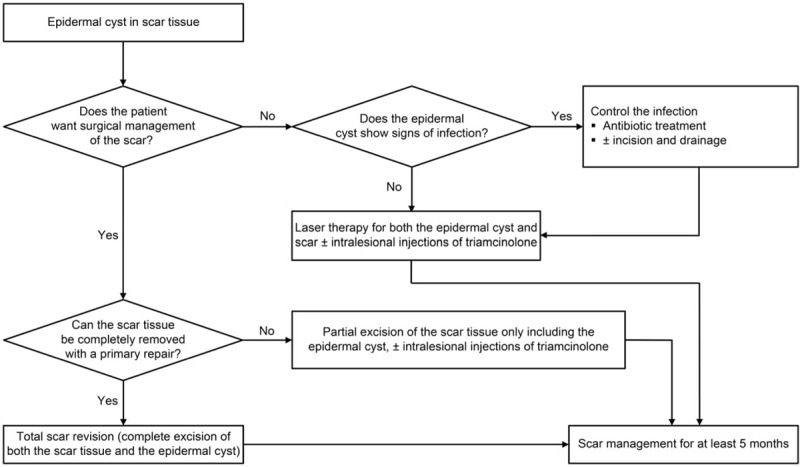
An algorithm for managing epidermal cysts arising from scar tissues.

Each treatment modality of ECs and scars is well known, as aforementioned. However, an EC arising from scar tissue is uncommon and the ideal treatment has not been established. We have developed a simple algorithm for the treatment of ECs arising from scar tissues, considering the circumstances that can occur as much as possible (Fig. 4). Several points should be considered in relation to the management of this condition. First, surgeons and patients must decide whether both the EC and the scar tissue should be completely removed. Second, consideration should be given to the options chosen for the management of the EC. Third, postoperative scar management is necessary to prevent the recurrence of hypertrophic and keloid scars. Using this guidance and our devised algorithm, ECs arising from scar tissues could be successfully treated.

## Acknowledgment

The authors thank Editage (www.editage.com) for English language editing and publication support.

## Author contributions

**Conceptualization:** Kyu Nam Kim.

**Data curation:** Hae Woong Lee, Chang Gyun Kim, Ji Sun Song, In Chang Koh, Hoon Kim.

**Formal analysis:** Kyu Nam Kim.

**Methodology:** Kyu Nam Kim.

**Supervision:** Hae Woong Lee, In Chang Koh, Hoon Kim, Kyu Nam Kim.

**Writing – original draft:** Hae Woong Lee, Chang Gyun Kim, Ji Sun Song, Kyu Nam Kim.
